# Chemoprevention of Head and Neck Cancer by Green Tea Extract: EGCG—The Role of EGFR Signaling and “Lipid Raft”

**DOI:** 10.1155/2011/540148

**Published:** 2011-01-02

**Authors:** Muneyuki Masuda, Takahiro Wakasaki, Satoshi Toh, Masahito Shimizu, Seiji Adachi

**Affiliations:** ^1^Department of Otorhinolaryngology and Head and Neck Surgery, Kyushu Koseinenkin Hospital, 2-1-1, Kishinoura, Nishiku, Kitakyushu 806-8501, Japan; ^2^Department of Otorhinolaryngology, Graduate School of Medical Sciences, Kyushu University, 3-1-1 Maidashi, Higashiku, Fukuoka 812-8582, Japan; ^3^Department of Gastroenterology/Internal Medicine, Graduate School of Medicine, Gifu University, 1-1 Yanagido, Gifu 501-1194, Japan; ^4^Deptartment of Pharmacology, Graduate School of Medicine, Gifu University, 1-1 Yanagido, Gifu 501-1194, Japan

## Abstract

Over the past decade dose-intensified chemo-radiotherapy or molecular targeted therapy has been introduced into the treatments of head and neck squamous cell carcinoma (HNSCC) to improve the outcomes of this dismal disease. However, these strategies have revealed only limited efficacy so far. Moreover, the frequent occurrences of second primary tumor further worsen the prognosis of patients. In this context, early detection and chemoprevention appear to be a realistic and effective method to improve the prognosis as well as quality of life in patients with HNSCC. In this short paper, we discuss the potential of green tea extract, (-)-epigallocatechin-3-galate (EGCG) in HNSCC chemoprevention, focusing on two aspects that are provided recently: (1) evidence of clinical efficacy and (2) unique biological effects on “lipid raft” that emerged as an important platform of numerous biophysical functions, for example, receptor tyrosin kinases (RTKs) signalings including epidermal growth factor receptor (EGFR), which play critical roles in HNSCC carcinogenesis.

## 1. Introduction

Head and neck squamous cell carcinoma (HNSCC), the sixth most common cancer worldwide, often generates from critical organs including the larynx, pharynx, oral cavity, and tongue that play indispensable roles in social, respiratory, communicative, and nutritional functions [1]. Surgical intervention for these organs often leads to a considerable impairment of the patient's quality of life (QOL), albeit recent remarkable progresses in reconstructive surgery. Accordingly, the intensity of conventional DNA-damaging therapies (i.e., irradiation and chemotherapy) has been strengthened to the upper limit of human tolerance of acute toxicities during the last decade [2]. Short-term results of these treatments seem to be promising. However, it is still under debate whether these dose-intensified types of protocols would lead to the long-term overall survival as well as “functional” organ preservation, because these protocols occasionally cause considerable complications (e.g., requirement for feeding tube due to severe laryngeal and pharyngeal dysfunction) and potential treatment-related death [2–4]. Ongoing molecular targeted therapies in HNSCC revealed only marginal effects so far [5]. In addition, the frequent occurrences of second primary tumor further worsen the prognosis of patients with HNSCC [1, 6]. As a result, the dishonorable phrase that is routinely used in the *Introduction* of HNSCC studies: “Despite recent advancements in treatment modalities, the overall survival and QOL of patients with HNSCC have not improved significantly over the past decade” still holds true, especially for patients with advanced stage. In view of these findings, early detection and chemoprevention appear to be realistic and effective method to improve the prognosis as well as QOL of patients with HNSCC.

## 2. Evidence and Perspective of EGCG in Chemoprevention

**Figure 1:**
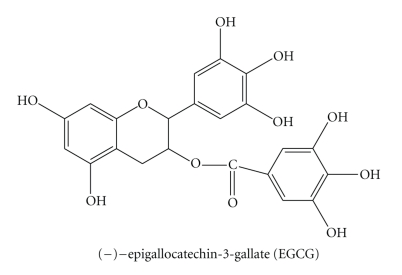
Structure of EGCG.

 As indicated by a recent review, we have witnessed remarkable progresses in the chemoprevention research in HNSCC [6]. A variety of natural and synthetic compounds have been shown to exert chemopreventive effects on HNSCC. Among them, a major active component of green tea extract, (-)-epigallocatechin-3-galate (EGCG), seems to be one of the most promising compounds that displays tumor suppressive effects on animal carcinogenesis model, mouse xenograft model, and a variety of cancer cell lines [7]. Figure 1 demonstrates the chemical structure of EGCG. Despite these substantial experimental data, there has been a longstanding question about the clinical efficacy of EGCG, because in a majority of *in vitro* studies, EGCG exhibits biological functions at relatively higher concentrations compared to the peak plasma concentrations obtained in individuals after administrating an oral dose of EGCG or decaffeinated green tea extract (<1 *μ*M) [8, 9]. However, recent studies provided evidence that administration of EGCG indeed has potential to reverse the process of carcinogenesis in patients with HNSCC or other human malignancies. In a phase II trial, Tsao et al. examined the effects of administration of green tea extract (GTE) capsule that contains 13.2% of EGCG for 12 weeks, three times a day at the dosage of 0 (placebo), 500, 750, or 1000 mg/m^2^/day on 41 patients with high-risk oral premalignant lesion. They found that two high dose arms (750 and 1000 mg/m^2^) revealed significantly (*P* = .03) higher response rates (58.8%) than 500 mg/m^2^ (36.4%) or placebo (18.2%) [10]. The group of Shimizu, who is one of the authors of this paper, demonstrated that administration of 500 mg of GTE tablet that contain 52.5 mg of EGCG three times a day (total 1500 mg/day) for 12 months significantly (*P* < .05) inhibited the incidence of second metachronous colon adenoma in patients who underwent endoscopic polypectomy, thus 31% in control arm versus 15% in the GTE treated arm [11]. Patients with high-grade prostate intraepithelial neoplasia received either placebo or 200 mg of GTE capsule that contain 51.8% of EGCG three times a day (total 600 mg/day) for 12 months. The GTE group displayed significantly (*P* < .01) lower incidence (3.0%) of prostate cancer compared to the placebo group (30.0%) [12]. No serious adverse effects were observed in any of these trials. Collectively, these studies indicate that administration of 50–200 mg of EGCG three times a day for 12 months appears to be safe and clinically effective protocol. Thus, the setting appears to be ideal for validating the clinical efficacy of EGCG in a larger-scale chemoprevention study. 

## 3. Diverse Molecular Target of EGCG

 A rapidly increasing number of mechanistic studies have revealed that in addition to the antioxidant effect, EGCG inhibits tumor development and progression by modulating wide spectrum of molecular targets. Those include RTKs: epidermal growth factor receptor (EGFR), erbB2/Her2, erbB3/Her3, erbB4/Her4, vascular endothelial growth factor receptor (VEGFR), platelet derived growth factor receptor (PDGR), insulin-like growth factor receptor (IRGFR) and hepatocellular growth factor receptor (HGFR), mitogen activated protein kinase (MAPK), proteasomes, matrix metro proteases (MMPs), cyclin-dependent kinases (CDKs), p53, DNA methyltransferase, Bcl-2, VEGF, reactive oxygen species (ROS,) 67 kDa laminin receptor (67LR), vimentin, phosphatidylinositol-3-kinase (PI3K)-Akt, NF-*κ*B, signal transducers and activators of transcription 3 (Stat3), and AP-1. These surprisingly diverse interactions between EGCG and target molecules or pathways are summarized in a recent comprehensive review [7]. In this short paper, we will mainly discuss the effects of EGCG on receptor tyrosin kinases (RTKs), especially EGFR, and their cell surface vessel, “lipid rafts,” that have emerged as a critical target of EGCG as well as an essential platform for signal transduction. 

## 4. The Role and Mechanism of EGFR Activation in HNSCC Carcinogenesis

**Figure 2:**
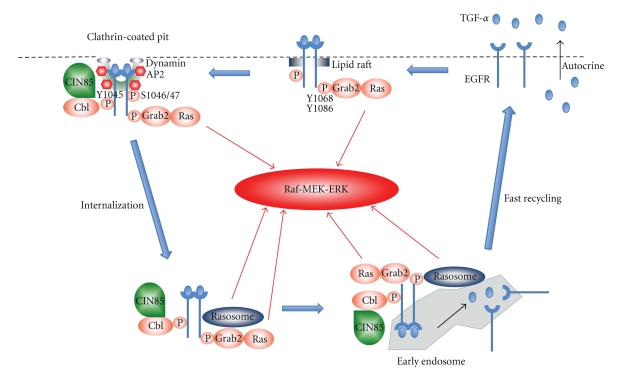
A proposed mechanism of TGF-*α*/EGFR/ras-MAPK activation loop in HNSCC. TGF-*α* binding to EGFR leads to dimerization and phosphorylation on lipid rafts. Phosphorylation of Y1068 and Y1086 is required for Grab2 binding and consequent ras activation. Activated EGFR dimmer is internalized via clathrin-coated pit. Cbl and CIN85 (overexpressed in 40% of HNSCC samples) are recruited at pY1045 and facilitate EGFR internalization. Phosphorylation of S1046/1047 is also necessary for EGFR internalization, albeit the precise role remains elusive. Recent evidence suggests that TGF-*α*-bound EGFRs signal in the cytosol, activating ras-MAPK cascade. Ras-enriched small cytosolic nanoparticles, “rasosomes,” might contribute to this signaling. Internalized TGF-*α*-bound EGFRs are sorted to early endosome. TGF-*α* dissociates from EGFR in the acidic environment of endosome. Free EGFR is recycled back via fast recycling back pathway to plasma membrane and is activated by TGF-*α* in a autocrine manner, resulting in constitutive activation of TGF-*α*/EGFR/ras-MAPK.

**Figure 3:**
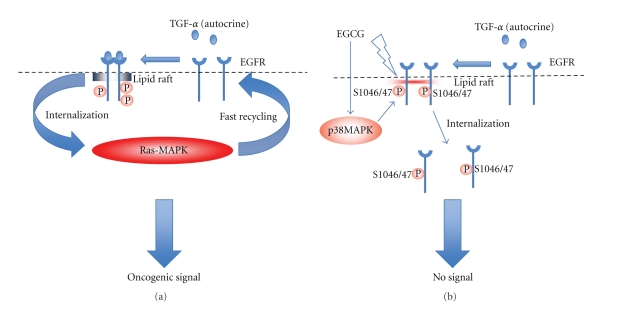
(a) Summary of TGF-*α*/EGFR/ras-MAPK activation loop in HNSCC (for more detail see Figure 2). (b) Inhibitory effect of EGCG on EGFR activation. EGCG alters organization of lipid rafts and promotes internalization of nonactivated monomer EGFR into cytosol through phosphorylation of EGFR at serine 1046/1047 by p38MAPK. As a result, EGCG causes a marked reduction of phosphorylated EGFR and thereby inhibits EGFR signaling that is prominent in HNSCC.

 In 1990s, Grandis et al. demonstrated that EGFR and its ligand transforming growth factor-*α* (TGF-*α*) mRNA were overexpressed in approximately 90% of HNSCC tumors, and overexpression of these two proteins was significantly associated with poor prognosis of patients with HNSCC [13, 14]. To date, numerous studies have revealed that EGFR signaling orchestrates tumor development and progression by activating several downstream signal transduction pathways including MAPK, Stat3, PI3K-Akt-mTOR, protein kinase C (PKC), and NF-*κ*B [15–17]. Several mechanisms have been postulated to explain aberrant EGFR signaling in human malignancies [15, 16]. Those include (1) receptor overexpression, (2) autocrine or paracrine activation by ligand overexpression or excessive ligand cleavage from cell surface by ADAM family metalloprotease, (3) gene amplification, (4) ligand independent activation through other receptor systems (e.g., erbB2), (5) constitutive active EGFR mutants: somatic activating mutation or truncated EGFRvIII, and/or (6) loss of negative regulation (e.g., EGFR degradation). Despite EGFR is one of the most extensively investigated molecules in HNSCC pathogenesis, the predominant mechanism of EGFR activation remains elusive. The EGFR gene amplification was observed only in 7 out of 33 patients with HNSCC, and intriguingly this amplification did not lead to protein overexpression [18]. However a recent study demonstrated that 49 out of 145 oral premalignant lesions displayed EGFR protein overexpression which was associated with relatively high incidence (41%) of EGFR gene copy [19]. Thus, the correlation between the EGFR gene amplification and protein expression is still under debate. The possibility of excessive cleavage of TGF-*α* and amphiregulin was demonstrated in HNSCC cell lines [20] but is not confirmed in clinical samples. The reported rates of somatic mutation of EGFR in HNSCC range as low as 7-8% [21–23]. Sok et al. found EGFRvIII expression in 42% of 33 HNSCC samples employing both immunohistochemical and RT-PCR assays [24]. In contrast, Yang et al. reported only 15% of EGFRvIII positive rates in 39 Chinese laryngeal cancer [25]. Interestingly, in 82 HNSCC samples from Japanese population, EGFR vIII was not detected [23]. Here again, the role of EGFRvIII in HNSCC is still controversial. The mechanism of EGFR internalization, degradation and recycling is a quite essential aspect that is closely associated with EGFR signaling [26]. However, there were few reports, which investigated this mechanism in HNSCC. We recently examined the role of multiadaptor protein c-Cbl interacting protein of 85 kDa (CIN85) [27] in HNSCC focusing on its role in EGFR signaling pathway [28]. In this study, we found that (1) CIN85 significantly facilitates EGFR internalization without apparently altering the levels of phosphorylated EGFR protein (i.e., EGFR signal intensity), consistent with the theory that TGF-*α* bound EGFRs are mainly sorted to the recycling-back pathway escaping from degradation, while a majority of EGF-bound EGFRs are processed via the degradation pathway [26], (2) TGF-*α* bound EGFR receptor signals in the cytosol as well as on plasma membrane, activating ras-MAPK cascade (ras-enriched small cytosolic nanoparticles, “rasosomes,” might contribute to this signaling [29]), (3) CIN85 silencing, therefore, inhibits EGFR internalization and activation of ras-MAPK cascade, and (4) CIB85 overexpression observed in 40% of HNSCC tumor samples contributes to the development of EGFR/ras-MAPK activation loop (Figures 2 and 3(a)). This model, at least in part, accounts for the reason why not EGFR but TGF-*α* is prominent mitogen in HNSCC development and progression. Nevertheless, it should be emphasized that the mechanism that causes TGF-*α* and EGFR overexpression in HNSCC remains elusive, although almost 20 years have passed since Grandis et al. [13, 14] first reported the significance of this overexpression. 

## 5. EGCG Inhibits EGFR: The Role of Lipid Raft

 Irrespective of the mechanisms which underlie EGFR activation in HNSCC, it was discovered by Liang et al. that EGCG can directly inhibit the binding of EGF to EGFR and thereby inhibits EGFR signaling [30]. Consistent with this finding, we first provided evidence that EGCG indeed inhibits EGFR activation in HNSCC cell line that displays autocrine activation of EGFR by TGF-*α*  [31]. We further examined the effect of EGCG on erbB2/Her2 employing HNSCC and breast cancer cell lines, and found that EGCG can inhibit the erbB2/Her2 activation, demonstrating the first example of erbB2/Her2 inhibition by EGCG in human malignancies [32]. Thereafter, we and other investigators confirmed the inhibitory effects of EGCG on other RTKs including erbB3/Her3, erbB4/Her4, IGFR, PDGFR, FGFR, and VEGFR employing a variety of cancer cell lines derived from different organs [33–38]. These ubiquitous inhibitory effects of EGCG on a series of RTKs, combined with the fact that the inhibitory effect of EGCG on EGF/EGFR binding was found only in a subcellular system [30], raised a question that EGCG might inhibit RTKs by a more general mechanism.

 Due to recent remarkable progresses in methods to analyze the structure, dynamic assembly, and function of nanoscale molecules, it is beginning apparent that cell membranes play critical roles in coordinating a variety of biochemical reactions including RTKs signal transduction [39–42]. Nanoscale transient membrane domains, “lipid rafts,” that are enriched with cholesterol, glycosphingolipids, glycosylphosphatidylinositol-anchored protein, caveolin-1, and signaling molecules, function as signaling platforms [39–41]. Among the RTKs, the interaction of EGFR with lipid rafts is most well understood [39]. Activation of EGFR by ligand and consequent signal transduction begins at lipid rafts, while its internalization occurs at clathrin-coated pit by further recruiting the E3 ubiquitin ligase Cbl, CIN85, and endorphins. The role of CIN85 in EGFR signal transduction in HNSCC was discussed in the previous section. These observations made us to hypothesize that EGCG might inhibit the activation of EGFR or other RTKs by altering the formation of lipid rafts.

So far through a series of three studies [43–45] we found that (1) EGCG alters lipid organization on the plasma membrane, (2) EGCG promote the internalization of nonactivated monomer EGFR into cytosol, thus, inhibiting the activation of EGFR by EGF, (3) as a result, treatment with EGCG causes marked reduction of phosphorylated (activated) EGFRs, that are otherwise preferentially present in lipid rafts, (4) EGCG-induced EGFR internalization requires neither the binding of c-Cbl to EGFR nor a phosphorylation of EGFR at tyrosine residue, suggesting that this internalization is mediated by a different mechanism that is observed in EGF-treated cells, and (5) phosphorylation of EGFR at serine1046/1047 mediated by p38MAPK is essential for EGCG-induced EGFR internalization (Figure 3(b)). 

In parallel with our findings, a Japanese research group discovered that 67LR, a constituent protein of lipid rafts, is an important binding target of EGCG [46]. 67LR is a nonintegrin laminin receptor, which is overexpressed on cell surface of various types of tumors, and the expression level of this protein strongly correlates with the aggressive phenotypes of tumor, albeit its role in HNSCC carcinogenesis is not investigated so far [47, 48]. Intriguingly, the predicted *K*_*d*_ value for the binding of EGFG to 67LR is as low as 40 nM, and physiological concentration of EGCG indeed inhibits the growth of human lung cancer cell line in a 67LR-dependent manner [46]. Although it is not clear whether the above-mentioned inhibition of EGFR by EGCG is relevant to 67LR, this finding also provides evidence that EGCG exerts antitumor effects through the interaction with lipid rafts protein. 

As mentioned in the “*Introduction,”* the EGFR targeted therapies, either used alone or in combination with radiation, have shown only limited efficacy so far, albeit its significant role in HNSCC carcinogenesis [5]. One of possible explanations for this insensitivity is that other growth factors or cytokines can surrogate EGFR signaling and activate downstream signal cascades including MAPK, Stat3, and PI3k-Akt. Then, HNSCC can relatively easily escape from EGFR dependency. However, Zhang et al. demonstrated that EGCG can synergistically enhance the growth inhibitory effects of EGFR tyrosine kinase inhibitor, erlotinib, both *in vitro* and in animal xenograft models employing HNSCC cell lines [49]. Consistent with our findings, treatment of EGCG significantly enhanced EGFR internalization that was not observed with treatment of erlotinib alone. Thus, they speculate that this internalization and consequent degradation of EGFR might be a major mechanism that accounts for this synergistic interaction. However, given the fact that a majority of growth factors or cytokines, which can surrogate EGFR signaling, utilize lipid rafts as signaling platforms [48], this synergistic interaction might be caused through the general inhibitory effects of EGCG on these growth factors or cytokines in lipid rafts. Thus, the addition of EGCG to RTKs targeting therapies might be an attractive strategy, which leads to the prevention of drug-tolerance, as is frequently observed in several clinical settings. 

## 6. Conclusions

 Considering the tantalizingly marginal improvement in the treatment outcomes of patients with HNSCC, it is urgent and critical to develop novel strategy based on early detection and chemoprevention. Among numerous putative chemopreventive agents, EGCG appears to be one of the most promising natural compounds based on accumulated data and, in particular, two novel findings provided recently: (1) clinical efficacy and (2) unique biological effects on lipid rafts that are an important platform of numerous biophysical functions including RTKs signalings. A larger-scale clinical study of EGCG is highly recommended.
